# Embedding patient voice within European healthcare: achievements and future directions of the health outcomes observatory

**DOI:** 10.1186/s41687-026-01221-y

**Published:** 2026-10-08

**Authors:** Preston Long, Rahim Lalji, Margaret R. Andrews, Afroditi Avgerinou, Olga Alekseeva, Francesco Patalano, Nataliya Kemenyash, Lisa R. Otto, Gemma Galan, Linetta Koppert, Meni Styliadou, Tanja Stamm

**Affiliations:** 1 Institute for Outcomes Research, Medical University of Vienna, Vienna, Austria; 2 Novartis Pharmaceuticals UK, London, UK; 3 Pfizer Hellas, Thessaloniki, Greece; 4 AstraZeneca, Cambridge, UK; 5 Novartis, Basel, Switzerland; 6 Takeda (Switzerland), Zurich, Switzerland; 7 Charité - Universitätsmedizin Berlin, Berlin, Germany; 8 Vall d’Hebron Institut de Recerca, Barcelona, Spain; 9 Erasmus MC Cancer Institute, Rotterdam, Netherlands

## Introduction

Europe has a rich history of collecting real-world data. Although sources are multiple, collection efforts are fragmented, lack standardization, and ownership remains unclear [1]. Furthermore, a key wellspring of information – the patient voice – is often underutilized. Without dedicated patient-reported outcomes (PRO) registry infrastructures, patient-reported data risk remaining fragmented, inconsistently collected, and difficult to reuse for care improvement, HTA, regulatory decision-making, and policy assessment. Previous reviews have shown only a minority of real-world evidence (RWE) studies include PROs [2]. These data are drivers for achieving the vision of the European Health Data Space (EHDS) [3]. PROs provide value for symptom monitoring, healthcare provider (HCP) -patient communication, health technology assessment (HTA), quality improvement and policy assessment [4]. In 2020, the Innovative Medicines Initiative (IMI), a EU public-private partnership, launched the Health Outcomes Observatory (H2O) project to address these gaps [5]. In this commentary, we (1) describe the establishment, pan-European structure, and governance of the H2O IMI project; and (2) discuss impacts, lessons learned, and future directions.

## H2O vision and concept

H2O was established to strengthen the role of the patient voice in routine care by supporting the standardized collection and use of PROs [5]. Furthermore, H2O aimed to provide data infrastructures that support cross-sector and pan-European aggregation of data for policymaking and research. To address selected limitations in current RWE generation in Europe, a multistakeholder consortium of 26 academic, public, and private partners was established in 2020.

First, a governance model for data use and sharing across Europe was developed. Pragmatic core-outcomes sets (COS) were subsequently established through Delphi methodology to standardize data collection across countries for both PROs and clinical variables. National observatories (NOs), not-for-profit legal entities, governed by independent boards were set up in Austria, Netherlands, Germany, and Spain. A Pan-European observatory (PEO) was established in Denmark for harmonization of data and strategy. Patient-level data collection from NOs began in 2023.

## Establishment of national observatories

Table 1 provides an overview of the tier 1 established national observatories.

**Table 1:** Overview of H2O national observatories

Location; Founding Date	Vienna, Austria; 09/09/2022	Berlin, Germany; 30/05/2023	Rotterdam, Netherlands; 22/07/2022	Barcelona, Spain; 13/05/2022
Observatory Name	Health Outcome Observatory Austria -- Österreichisches Observatorium für Gesundheitsergebnissen	Health Outcomes Observatory – Germany (“H2O e.V.”)	H2O Nederland	Observatorio H2O España
Constituencies Represented (n)	Society overall (*n* = 1); Patient organizations (*n* = 1); Medical professionals; (*n* = 1); Researchers (*n* = 1); Regulatory authorities (*n* = 1); Private sector (*n* = 1); Technology providers (*n* = 1)	Patient organizations (*n* = 2); Healthcare providers (*n* = 2); Civil society (*n* = 1); Research and academia (*n* = 3); Private sector (*n* = 1); Public sector (*n* = 3)	Patient organisations (*n* = 1); Medical professionals (*n* = 4); Society overall (*n* = 1); Regulatory agency and/or HTA authority (*n* = 1); Private sector (*n* = 5)	Civil Society, Research & Academia (*n* = 4); Patient organizations (*n* = 2); Healthcare providers (*n* = 5); Private sector (*n* = 3)
Governance structure	• NO comprised of Board of Directors; General Assembly with representation of all constituencies; auditors. • NO supports data platform hosted by the Austrian National Public Health Institute and the Medical University of Vienna as data co-controllers.	• Non-profit association comprised of a General Assembly (persons and institutions) and a Board of Directors representing all stakeholder groups. One managing director is elected by the Board of Directors.	• Non-profit association comprised of a General Assembly (natural persons and institutions) and a Board of Directors and Supervisory Board representing all constituencies	• Non-profit association comprised of a General Assembly and a Board of directors representing all constituencies.
Technical solution	Country-specific, EHR-linked PRO platforms with secure local storage and patient agreement under federated governance, unified by a Pan-European Observatory for standardisation, compliant sharing, and scalable cross-country research.			
Sites & data sources	4 clinics; 1 registry Italian data San Raffaele University	3 clinics, 1 registry, 1 patient organization	2 clinics, 3 patient organizations	3 hospitals; 9 clinics
Alignment with national healthcare systems	Complies with national health policies and privacy regulations; integrated with hospital networks and registries; contributes data for public health reporting and research; collaborates with scientific societies and patients’ organizations.			
Collaboration & interoperability	Regular meetings (online and in-person), events and stakeholder outreach efforts; special topic working groups; use of common data standards (I.e., OMOP CDM) and outcome sets; interoperable platforms; collaborative projects (I.e., registry integration).			

## The pan-European health outcomes observatory

The PEO aims to link national observatories using a governance model which was developed within the H2O project and provide coordination and guidance on digitally measurable patient outcomes in routine care. It also develops a business strategy to promote the financial sustainability of its members, and supports the establishment of new observatories. The PEO operates under a board structure. The PEO board includes representation from National Observatories, patient organizations and regulators / HTA authorities.

## Disease areas and patient recruitment

H2O’s initial focus areas were Diabetes, Inflammatory Bowel Disease, and Cancer (metastasized breast and lung). These areas were selected due to their high prevalence and chronic nature. Patient Advisory Boards ensured continuous patient engagement from the outset. Multi-stakeholder Delphi exercises for each indication including patients’ voices resulted in the H2O core outcome sets, which were subsequently adopted pragmatically (or in full) across the four countries [6–10]. The H2O model was designed to allow flexibility in technology solutions, with a range of applications used for PRO collection and outcome visualization including patient facing dashboarding. Since 2023, 2000 patients participated in H2O with most of the patients been recruited in Austria (77%).

## Challenges and lessons learned

The key lesson has been the influence of diverse organizational, legal, and national contexts at site-, and country-levels. The experience of H2O highlights that PRO registries require more than technical platforms: they depend on workflow integration, clinician engagement, patient trust, clear governance, and visible value for participants. Local variations required ongoing adaptations and adjusted timelines. Complexities around data standardization, interoperability, workflow integration, and data sharing demand careful navigation, and both systemic change and strong stakeholder, in particular patients themselves, buy-in remain critical. Despite these challenges, early implementation suggests that routine PRO collection is feasible in selected settings, although continued evaluation is needed to understand uptake, representativeness, and long-term sustainability. Scaling to additional sites will require continued adaptation, and collected data must be made actionable in the clinic in a timely manner to maintain patient and HCP engagement. Safeguarding patient trust and engagement in data sharing and standardization will be essential for sustainable adoption and meaningful impact. PRO registries are only useful if patients, clinicians, and data users trust the infrastructure, understand its purpose, and see value from continued participation.

## Impact and future directions

H2O’s contributions include continuous stakeholder engagement and co-creation, development of the core outcome sets [6–10], procurement, and refinement of technology solutions, data standardization, patient-facing dashboarding, thought leadership activities, and expansion to new centers and indications. The H2O Patient Agreement was designed to strengthen patient control by providing multiple options for potential data use while supporting both primary and secondary research. H2O’s next phase builds on work already underway to sustain the observatories and translate their pragmatic core outcome sets into lasting value for care and research. Continued longitudinal data collection will support routine consultations and secondary research on symptom and quality-of-life trajectories, variation across settings, and sustained patient participation. The Pan-European H2O is a partner in a new IHI project, while the national H2O observatories are also involved in local projects that build on and further apply the H2O approach, thus extending opportunities to apply its methods and infrastructure in additional clinical contexts. Work is also underway to map patient-reported outcome measures (PROMs) to the Observational Medical Outcomes Partnership (OMOP) Common Data Model. The next steps are to validate these mappings across observatories and share reusable specifications that support analyses combining patient-reported and clinical data.

Ongoing collaboration with the European Medicines Agency is exploring how routinely collected PROs can inform regulatory evidence, including through lung cancer and diabetes case studies. These applications will assess data completeness, representativeness, and interpretability against specific research questions. In parallel, engagement with health technology assessment bodies should examine potential contributions to Joint Clinical Assessment. The roadmap therefore links sustained collection and harmonization with secondary analyses, methodological validation, and evaluation of defined regulatory and assessment applications. Patient involvement in research priorities and data governance, alongside feedback to participating services, will remain central to this work.

## Conclusion

H2O represents a multi-country effort to jointly standardize, collect, and analyze PROs and clinical outcomes, contributing to the European dialogue on PROs and patient-centered healthcare. Long-term success will depend on sustained collaboration, continued patient access to meaningful insights from their own data and alignment with European health data and digital health initiatives.

## Supplementary Information

Below is the link to the electronic supplementary material.

Supplementary Material 1

## Data Availability

No datasets were generated or analysed during the current study.
