# Supplementary material for: Embedding patient voice within European healthcare: achievements and future directions of the health outcomes observatory
Source: J Patient Rep Outcomes. 2026 Oct 8;10:184. doi: 10.1186/s41687-026-01221-y (PMC13650199; doi:10.1186/s41687-026-01221-y)
Supplement: Supplementary file 1 [file 41687_2026_1221_MOESM1_ESM.docx]

**Table 1** provides an overview of the tier 1 established national observatories.

**Table 1: Overview of H2O National Observatories**

| **Location; Founding Date** | Vienna, Austria; 09/09/2022 | Berlin, Germany; 30/05/2023 | Rotterdam, Netherlands; 22/07/2022 | Barcelona, Spain; 13/05/2022 |
| --- | --- | --- | --- | --- |
| **Observatory Name** | Health Outcome Observatory Austria -- Österreichisches Observatorium für Gesundheitsergebnissen | Health Outcomes Observatory – Germany (“H2O e.V.”) | H2O Nederland | Observatorio H2O España |
| **Constituencies**  **Represented (n)** | Society overall (n=1); Patient organizations (n=1); Medical professionals; (n=1); Researchers (n=1); Regulatory authorities (n=1); Private sector (n=1); Technology providers (n=1) | Patient organizations (n=2); Healthcare providers (n=2); Civil society (n=1); Research and academia (n=3); Private sector (n=1); Public sector (n=3) | Patient organisations (n=1); Medical professionals (n=4); Society overall (n=1); Regulatory agency and/or HTA authority (n=1); Private sector (n=5) | Civil Society, Research & Academia (n= 4); Patient organizations (n=2); Healthcare providers (n=5); Private sector (n=3) |
| **Governance structure** | - NO comprised of Board of Directors; General Assembly with representation of all constituencies; auditors. - NO supports data platform hosted by the Austrian National Public Health Institute and the Medical University of Vienna as data co-controllers. | - Non-profit association comprised of a General Assembly (persons and institutions) and a Board of Directors representing all stakeholder groups. One managing director is elected by the Board of Directors. | - Non-profit association comprised of a General Assembly (natural persons and institutions) and a Board of Directors and Supervisory Board representing all constituencies | - Non-profit association comprised of a General Assembly and a Board of directors representing all constituencies. |
| **Technical solution** | Country-specific, EHR-linked PRO platforms with secure local storage and patient agreement under federated governance, unified by a Pan-European Observatory for standardisation, compliant sharing, and scalable cross-country research. | | | |
| **Sites & data sources** | 4 clinics; 1 registry Italian data San Raffaele University | 3 clinics, 1 registry, 1 patient organization | 2 clinics, 3 patient organizations | 3 hospitals; 9 clinics |
| **Alignment with national healthcare systems** | Complies with national health policies and privacy regulations; integrated with hospital networks and registries; contributes data for public health reporting and research; collaborates with scientific societies and patients’ organizations. | | | |
| **Collaboration & interoperability** | Regular meetings (online and in-person), events and stakeholder outreach efforts; special topic working groups; use of common data standards (I.e., OMOP CDM) and outcome sets; interoperable platforms; collaborative projects (I.e., registry integration). | | | |
